# Measuring Resilience Across Participating Regions in the UPRIGHT EU Horizon 2020 Project: Factor Structure and Psychometric Properties of the Resilience Scale for Adolescents

**DOI:** 10.3389/fpsyg.2021.629357

**Published:** 2021-02-17

**Authors:** Frederick Anyan, Roxanna Morote, Carlota Las Hayas, Silvia Gabrielli, Iwona Mazur, Dora Gudrun Gudmundsdottir, Nerea González, Anna Królicka-Deregowska, Antoni Zwiefka, Anna S. Olafsdottir, Odin Hjemdal

**Affiliations:** ^1^Department of Psychology, Norwegian University of Science and Technology, Trondheim, Norway; ^2^Kronikgune Institute for Health Services Research, Barakaldo, Spain; ^3^Bruno Kessler Foundation, Trento, Italy; ^4^Daily Centre for Psychiatry and Speech Disorders, Wrocław, Poland; ^5^Wybrzeże L. Pasteura 1, Wroclaw Medical University, Wrocław, Poland; ^6^Directorate of Health, Reykjavík, Iceland; ^7^Osakidetza Basque Health Service, Barrualde-Galdakao Integrated Health Organisation; REDISSEC (Health Services Research on Chronic Patients Network), Barakaldo, Spain; ^8^Lower Silesian Voivodeship Marshal Office, Wroclaw, Poland; ^9^University of Iceland, School of Education, Reykjavik, Iceland

**Keywords:** Resilience Scale for Adolescents, cross-cultural validation, measurement invariance, psychometric properties, protective factors, UPRIGHT project

## Abstract

Resilience is the process and outcome of healthy adaptation despite significant adversity. Proliferation of research on the resilience construct has led to scientific concerns about the operationalization and measurement of resilience for assessment science and practice. Various studies that have investigated the psychometric properties and construct validity of the Resilience Scale for Adolescents (READ) have yielded inconsistent findings, which could partly be due to variations in the methodological approaches. This study investigated the factor structure and construct validity of the READ in four European regions participating in the Universal Preventive Resilience Intervention Globally Implemented in Schools to Improve and Promote Mental Health for Teenagers (UPRIGHT) project. Participants included adolescents aged 10–15 years from Spain (*n* = 391, females = 51%), Iceland (*n* = 379, females = 55%), Italy (*n* = 460, females = 55%), and Poland (*n* = 316, females = 51%). The five-factor model of the READ was similar across gender and participating regions. Construct validity of the READ was supported. After establishing construct separability, incremental validity was supported (except for the social competence subscale). The READ is a valid and reliable measure of protective factors involved in resilience and demonstrates promise for cross-cultural applicability. Recommendations for measuring resilience and validating the READ in future investigations are provided.

## Introduction

Systematic reviews of the global burden of mental health problems in young people suggest that anxiety and depression continue to increase even in the context of underreporting and detection (Gore et al., 2011; Bor et al., 2014). The situation in European countries is no different. The World Health Organization (WHO) European Region report on adolescent mental health indicated that anxiety and depression are among the top five causes of overall disease burden (World Health Organization, 2018). Schools are key settings for intervention programs to promote positive mental health (Ford and Finning, 2020). In a systematic review of resilience-enhancing and universally delivered school-based mental health promotion programs, the authors concluded that programs that focused on resilience and coping skills positively impacted students and helped them to manage daily stressors (Fenwick-Smith et al., 2018). Successfully delivered programs included key components that focused on teacher involvement, student engagement, participatory methods to engage students, and the use of multiple methods for program evaluation (Fenwick-Smith et al., 2018).

Resilience carries much promise for promoting adaptive mental health (Anyan et al., 2018; Anyan, 2019) and refers to the process of, or outcome of, successful adaptation despite adverse life circumstances that increase the probability of mental health problems (Masten, 2001). The UPRIGHT (Universal Preventive Resilience Intervention Globally Implemented in Schools to Improve and Promote Mental Health for Teenagers) project (Las Hayas et al., 2019) was designed as a resilience-enhancing program that incorporates an ecological framework of resilience by targeting the school environment, the school staff, family, and adolescents as active participants across five EU regions, namely, the Basque Country (Spain), Reykjavík (Iceland), Trentino (Italy), Lower Silesia (Poland), and Denmark (henceforth, intervention sites).^1^ Resilience has become an auspicious intervention initiative due to its promising results, being one of the most integrative concepts with an interdisciplinary approach and cost-effective implementation (Anyan, 2019). The proliferation of research on the resilience construct has led to growing scientific concerns about the discrepancies that exist in operationalizing resilience for assessment science and practice (Luthar et al., 2000; Anyan, 2019). Consequently, measuring resilience requires rigorous reliability and validity tests (e.g., structural validity through measurement invariance across groups, contexts, and cultures) (Anyan, 2019).

When instruments are adapted to other languages for psychological assessment, it is important to investigate the measurement invariance of the translated versions. This is to ensure that the construct manifests in equivalent fashion, is measured similarly, and that the scale functions in the same way across the different groups, contexts, or cultures. For example, if the meaning and the way resilience manifests differ across the intervention sites and participants respond to the items of a resilience scale differently, it will impose restrictions on the generalizability of its findings. This may involve participants using different starting points to scale their responses despite being on the same level of the latent resilience construct. Such a finding would indicate that the observed indicators of the resilience scale is statistically non-equivalent across samples in the intervention sites, and therefore, conclusions about the project findings must take these differences into account. Measurement invariance analysis can pinpoint any sources of differences across a hierarchy of levels ranging from configural invariance to scalar invariance for cross-cultural comparison of scale means. To measure resilience reliably and validly is important as a first step toward expanded research, rigor in applied practice, fidelity to social policy, and informed preventive interventions (Anyan, 2019; Anyan et al., 2019). The goal of the current study was to investigate the operationalization of resilience by the Resilience Scale for Adolescents (READ) across participating regions in the UPRIGHT project.

### General Description of the READ

The READ is a copyrighted instrument that the authors grant permission to use following a request. READ measures central protective factors involved in resilience. It was developed based on its adult predecessor version (Resilience Scale for Adults, RSA) (Hjemdal et al., 2001; Friborg et al., 2003) through an extensive review of contemporary studies by assembling available empirical evidence. In complying with the resilience theoretical framework, the READ, like the RSA, covers the three overarching dimensions (positive personal factors, family environmental factors, and external social support) identified to be mutually involved as protective factors. These factors encourage and reinforce resilience processes or outcomes (Friborg et al., 2003; Hjemdal et al., 2006). The READ contains 28 positively phrased items organized into five rank-order Likert responses for easy interpretation and completion in adolescent samples. The factors contained in the READ are: (i) personal competence, (ii) social competence, (iii) structured style, (iv) social resources, and (v) family cohesion.

Personal competence assesses an individual's level of self-esteem, self-efficacy, self-acceptance, hope, determination, and realistic goal orientation to life as well as the ability to organize and plan. Social competence measures the ability to start a conversation in social settings, to be flexible and bring one's self into social encounters, good communication skills, extraversion, and cheerful mood. Structured style concerns the degree of preference for planning and structuring daily routines. Family cohesion measures familial shared values and family support structures as well as the family's ability to keep a positive outlook despite adverse circumstances. Finally, social resources assess the level of perceived access and availability of external social support networks outside the family, including relatives and friends (Hjemdal et al., 2006).

### Previous Psychometric Evaluations of the READ

Various studies have supported the construct validity of the READ and its subscales. They include negative correlations with measures of stressful life events, experience of bullying, anxiety, and depressive symptoms (Hjemdal et al., 2006; Anyan and Hjemdal, 2016, 2018; Kelly et al., 2017; Anyan et al., 2018; Moksnes and Haugan, 2018; Askeland et al., 2019). Others include positive correlations with sense of coherence and self-esteem (Moksnes and Haugan, 2018), safe neighborhood, enjoying family life, and informal help-seeking (Kelly et al., 2017) as well as participation in hobbies, team sports or athletic clubs, and active engagement in social interactions (Hjemdal et al., 2006). Following the seminal work in developing the READ, additional validation studies in Norway have been conducted in adolescent samples aged 18–20 years (*N* = 6,723; von Soest et al., 2010; *N* = 9,596; Askeland et al., 2019) and 13–18 years (*N* = 1,183; Moksnes and Haugan, 2018). Other validation studies have been conducted outside Norway, including adolescent samples aged 18–20 years from Italy (*N* = 472; Stratta et al., 2012), adolescents aged 12–17 years from Mexico (*N* = 840; Ruvalcaba-Romero et al., 2014), and 12–19 years old from Ireland (*N* = 6,030; Kelly et al., 2017).

All previous studies have supported a five-factor structure of the READ, although some studies have raised questions about inconsistencies in the original factor-item patterns (e.g., von Soest et al., 2010; Ruvalcaba-Romero et al., 2014; Moksnes and Haugan, 2018). These concerns have resulted in a 23-item five-factor solution (von Soest et al., 2010), a 26-item five-factor solution (Ruvalcaba-Romero et al., 2014), and even a 20-item five-factor solution (Moksnes and Haugan, 2018). Noticeably, concerns about potentially problematic items loading on factors other than the ones hypothesized may partly be due to the approach that some of the previous studies have used to validate the READ. As an initial validation of the READ, von Soest et al. (2010) conducted an exploratory factor analysis (EFA) to determine the number of factors to extract and found that the first five factors had eigenvalues >1. In a later separate confirmatory factor analysis (CFA) for each subscale using a random subsample (*n* = 1,000), the authors removed two items due to their high residual covariance. When a CFA was conducted on the remaining 26 items, three items were further removed for the same reason. The resulting measurement model was then cross-validated in the remaining sample. In their study, Stratta et al. (2012) conducted a principal component analysis (PCA) that identified six components. Subsequent analyses were forced to five- and four-component solutions due to problematic loadings. Then, using the same sample, separate confirmatory analyses were conducted on the PCA models as well as the revised five-factor solution obtained from the study by von Soest et al. (2010). Fit indices in the three CFA solutions were only modest, except the root mean square error of approximation (RMSEA, 0.051–0.064). Ruvalcaba-Romero et al. (2014) conducted an EFA with a resulting seven-factor model by removing two items. The authors indicated that one item related to “vital satisfaction” and participants could have interpreted the other item as “negative response.” Next, the authors then conducted a CFA on the 26- and 22-item factor solutions, removing further two items to improve the reliability coefficient of their corresponding subscales due to low standardized saturation (Ruvalcaba-Romero et al., 2014). Recently, Askeland et al. (2019) have discussed the READ from a theoretical and conceptual standpoint, noting some modifications to the factor structure. The authors found that the original 28-item five-factor model yielded a relatively poor fit, but found an improvement in a new 24-item five-factor revised model. Using exploratory structural equation modeling (ESEM), a different 28-item five-factor structure that divided personal factors into goal orientation and self-confidence was identified with better model fit.

Some strengths from previous studies include the use of large sample sizes and adolescents across a wider age range. Still, some of the previous studies partly show some methodological concerns in their construct validation approach. Firstly, the rationale for preceding construct validation of an existing measure by exploratory investigations (e.g., EFA) remains unclear. Using EFA is best suited for analyses of (i) unrestricted measurement models when the latent structure of a measure is unknown or (ii) when a confirmatory approach fails to reproduce an initial restricted measurement model (e.g., ESEM). When the latent structure is known, the analyses proceed in a confirmatory approach. Secondly, Moksnes and Haugan (2018) used a confirmatory approach on a split-half sample, and when the model fit was inadequate, the authors employed exploratory analysis, resulting in a 20-item five-factor model solution that retained the factors contained in the study by Hjemdal et al. (2006). It is, however, unclear why the authors did not investigate the measurement model using the whole sample when the model in the split half failed to reach adequate fit. Using a confirmatory approach and a measurement invariance framework, the study by Kelly et al. (2017) reported that the original five-factor model of the READ (Hjemdal et al., 2006) was adequately replicated, producing a more satisfactory fit than a hypothesized three-factor model (hypothesizing the three overarching factors of resilience), and support for the five-factor model was found across gender, school cycle, and distress levels. Thirdly, using PCA instead of a common factor analysis fails to account for random error variance or measurement unreliability in the indicators, thus resulting in attenuated component intercorrelations. This leads to statistically misleading conclusions about the factor structure since the components retain random error variance (Brown, 2015). Fourthly, previous studies have not mentioned whether positivity bias was inspected in the data and how it was dealt with.

The development of the READ recommended inspecting and remedying positivity bias (i.e., most people hold a positive view of themselves and the future) in the data that reflects a general tendency in the population when assessing resilience (Hjemdal et al., 2006). Normal samples encountering adversity can go on to preserve adaptive mental health, indicating that access to protective resources associated with resilience is common in a normal sample, consistent with Masten's (2001) claim of ordinary magic. Positivity bias means that, even though the response categories of READ are balanced with equal numbers of categories to the left and right sides of “(3) Average,” most responses fall on the extreme side of “(5) Totally agree.” Positivity bias can be amplified due to the items being phrased in a unified positive manner. Related to positivity bias is acquiescence bias, both of which can greatly affect the assumption of interval scale in READ's data (Soto et al., 2008). Acquiescence bias is more pronounced in younger children and adolescents, only decreasing by age 20, than in adults and accounts for seriously distorted and inconsistent factor structures (Soto et al., 2008). The presence of positivity bias and acquiescence bias in the READ data would therefore not be surprising as most Likert responses are vulnerable to these biases (Bishop and Herron, 2015). The consequence is that the assumption of linearity and the possibility of the items being an interval measurement would be greatly affected (Bishop and Herron, 2015; Brown, 2015). Hence, the factor structure of the READ could suffer a major setback, resulting in seriously distorted and inconsistent factor structures, especially when using normal theory continuous estimation (Soto et al., 2008). Without remedial estimation methods, the model estimation will inevitably produce factor solutions contaminated by artifacts of item extremeness and incorrect test statistics (Brown, 2015). Methods that estimate threshold parameters linking the underlying unobserved continuous characteristics (e.g., resilience) to the observed indicators can alleviate such problems (Brown, 2015; Finney et al., 2016; Svetina et al., 2020).

### Testing Measurement Invariance of the READ

Drawing conclusions about resilience processes and outcomes across different samples from different regions/countries and cultures requires investigating construct equivalence to determine what resilience means to the different groups, countries, cultures, and contexts. Other factors such as the quality of translation, sensitivity to cultural influences, and culture-specific knowledge about expressing relevant concepts related to good adaptation and promotion of mental health can affect the measurement properties of the READ. These factors necessitate testing READ's construct equivalence. Multigroup CFA measurement invariance (MGCFA MI) is probably the most popular method for investigating whether an instrument measures the intended latent construct equivalently across contexts or cultural groups. Measurement invariance of the READ instrument seeks to investigate whether the factor indicators measure the same resilience construct in the same way in the different intervention sites. The most important levels of measurement invariance required for cross-cultural comparisons of scale means are as follows:

- Equal factor structure (configural invariance—basic, very weak requirement)
- Equal factor loadings (metric invariance—weak requirement)
- Equal latent intercepts/thresholds (scalar invariance—strong requirement)

All tests should minimally support configural invariance, which simply examines whether the same factor structure (number of factors and the pattern of factor–item relations) can be assumed across all four countries. Support of configural invariance indicates that the READ measures similar latent resilience constructs; therefore, further restrictions can be imposed on the configural invariance model to test stronger degrees of construct equivalence. The next test, metric invariance, will detect whether factor loadings differ across the four countries. This is the most important test of MGCFA MI (Chen, 2007), which tests whether the relations (i.e., slopes) between the subscale items and the factors are parallel across intervention sites. If supported, this would mean that the samples from the four countries interpret the READ scale items similarly; hence, simple regression analyses based on raw scores may be used to conclude about cultural differences.

The threshold parameters that link the underlying continuous latent resilience construct to the observed response categories “(1) Totally agree” to “(5) Totally disagree” may differ, which the next test will detect. A stricter and often unrealistic invariance requirement is the scalar invariance, which requires equal intercepts or equal item thresholds for ordinal indicators (Millsap and Yun-Tein, 2004; Svetina et al., 2020). If supported, respondents from all intervention sites use the same starting point (intercept) for scaling their responses. In practice, respondents use the ordinal response categories comparably, allowing scale mean comparisons across intervention sites. At the scalar level, evidence for strong measurement invariance exists if the scalar invariance model fits the data as equally well as the metric invariance model, making group comparisons of latent means meaningful. The residual variances of items can also be constrained equal across intervention sites to test for strict measurement invariance. However, strict invariance is optional as it has no additional substantive information in applied research (Brown, 2015).

### Hypotheses

The five-factor structure of the READ was expected to replicate across intervention sites (support for configural invariance), as well as observing comparable factor loadings (support for metric invariance) and equal item intercepts/thresholds (support for scalar invariance). Support for the construct validity of the READ was expected by showing significantly positive correlations with measures of well-being focusing on positive aspects of mental health and health-related quality of life, but significantly negative with measures of perceived stress, anxiety, and depressive symptoms. Finally, it was expected that the READ will show incremental validity over and above the perceived stress and symptoms of anxiety when predicting depressive symptoms.

## Materials and Methods

### Participants and Procedure

Sample and effect size calculations for the UPRIGHT project were determined *a priori* and reported elsewhere (see Las Hayas et al., 2019). At the beginning of the UPRIGHT project in January 2018, pilot sites approached several schools in their regions. The UPRIGHT research project objectives, implementation, and evaluation procedures were presented to them. The schools which signed the letter of commitment were included to participate. Stratified randomization was used to divide schools into “blocks” according to their location and socioeconomic status. For sites having different school types (i.e., public and private), this feature was also considered. Within each “block,” schools were randomly assigned to the control or the intervention arm. Students completed the questionnaires if they attended/belonged to the classrooms designated for the UPRIGHT implementation and their parents/guardians gave authorization. The UPRIGHT intervention was received by all students in the classrooms. In the current study, participants were students between 10 and 15 years of age (*M* = 12.41, SD = 0.86) taking part in the first-wave implementation of the UPRIGHT intervention project in Basque Country/Spain (*n* = 391, females = 51%), Reykjavík/Iceland (*n* = 379, females = 5%), Trentino/Italy (*n* = 460, females = 55%), and Lower Silesia/Poland (*n* = 316, females = 51%). Participants who reported “Other” as gender included Iceland (*n* = 3), Italy (*n* = 1), and Poland (*n* = 3). These participants were excluded when investigating gender differences. Five participants (Italy, *n* = 3; Poland, *n* = 2) did not report their gender.

Ethical clearance was obtained by individual countries based on local regulations. The READ was translated into the languages of pilot sites using two stages of forward (from English to the local language) and backward (from local language to English) translations. Two bilinguals (i.e., native speakers of the language in each pilot site who are proficient in English) independently did the forward translations, then two new bilinguals blinded to the original version independently did the backward translations. All four versions from each pilot site were sent back to the copyright holders to compare the translations with the original and determine the best translation that retained the original meaning of the items. This is important since, very often, there are larger or smaller errors, misunderstandings, or adjustments that must be checked to ensure accuracy and to achieve homogeneity in the translation process.

### Instruments

The Resilience Scale for Adolescents (READ; Hjemdal et al., 2006) is a 28-item self-report scale using a five-point Likert scale, with all items positively phrased. Higher scores reflect a higher degree of resilience. The Warwick-Edinburgh Mental Well-Being Scale (WEMWBS; Tennant et al., 2007) is a 14-item self-report scale that assesses mental well-being focusing on positive aspects of mental health. WEMWBS items are positively phrased with a five-point Likert scale. Higher scores indicate higher levels of mental well-being. The KIDSCREEN-10 (Ravens-Sieberer et al., 2010) is a measure of health-related quality of life focusing on subjective health and well-being with a five-point response scale. Higher scores indicate better health-related quality of life. The shortest version of the Perceived Stress Scale (PSS-4; Cohen and Williamson, 1988) includes four items intended to measure global stress levels. Each of the items on the PSS-4 is measured on a five-point Likert scale, with higher scores indicating higher stress. The Generalized Anxiety Disorder (GAD-7; Spitzer et al., 2006) scale is a seven item self-report measure that assesses anxiety-related symptoms. All items are answered using a four-point Likert scale, with higher scores indicating more anxiety symptoms. The Patient Health Questionnaire (PHQ-9; Kroenke et al., 2001) is a nine-item self-report measure that assesses the frequency of depressive symptoms. All items are answered using a four-point Likert scale, with higher scores indicating more depressive symptoms.

### Statistical Analyses

SPSS 24.0 was used for descriptive statistics and correlational and linear regression analyses, while testing of measurement invariance and other SEM analyses were conducted in Mplus 7.4 (Muthén and Muthén, 1998-2015). Internal consistency was examined with coefficient alpha (which assumes tau equivalence) and coefficient omega (which accepts differences in tau, factor loadings). A uniform screening protocol was employed for all datasets (*N*_rawdata_ = 1,546) based on the widely accepted Mardia's multivariate skewness and kurtosis statistics. Mardia's skewness and kurtosis were calculated to assess the underlying assumption of multivariate normality. The READ scores were found to be non-normally distributed (Spain: Mardia's multivariate skewness, MS = 158.86, *p* < 0.001; Mardia's multivariate kurtosis, MK = 1,041.17, *p* < 0.001; Iceland: MS = 160.78, *p* < 0.001; MK = 1,078.54, *p* < 0.001; Italy: MS = 108.54, *p* < 0.001; MK = 971.87, *p* < .001; Poland: MS = 169.78, *p* < 0.001; MK = 1,016.17, *p* < 0.001).

Applied researches analyzing Likert response scales such as the READ either (i) ignore the categorical nature of the data and apply normal theory continuous estimation (e.g., maximum likelihood) or (ii) account for the categorical nature of the data by applying categorical estimation methods (e.g., diagonally weighted least squares). Frequency counts in the responses of the READ across all intervention sites revealed that, in some cases, the center option (3) Average and, in most cases, the highest right option “(5) Totally agree” were frequently used, indicating the presence of positivity and acquiescence biases. Various recommendations exist for using estimation methods that take the ordinality of Likert response scales (e.g., “Totally agree” to “Totally disagree” in the READ) into account when the data is non-normal (Brown, 2015) or there are at least four (Rhemtulla et al., 2012) or five (Finney et al., 2016) response categories. When there are six or more responses, either a continuous or a categorical estimation method can be used, but the results from categorical estimation are reported when there are differences in the results from the two estimation methods (Finney et al., 2016).

Due to potential consequences of positivity and acquiescence bias, evidence of non-normality across the data, and the fact that the READ item responses are categorized into five rank-order scales from “(1) Totally disagree” to “(5) Totally agree,” the data were estimated with the weighted least squares mean and variance adjusted (WLSMV). The WLSMV requirements for sample size are far less restrictive and it produces accurate test statistics, parameter estimates, and standard errors in CFA models with as low as *N* = 200 even when item extremeness is present (Brown, 2015). The choice of WLSMV was also guided by the fact that the family of weighted least squares estimation methods does not assume multivariate normality of factor indicators. In this way, the analysis overcomes normality assumptions (Brown, 2015; Kline, 2015), as well as producing accurate test statistics and less biased parameters, standard errors, and goodness-of-fit measures (Brown, 2015). Consistent with recommendations by Sass et al. (2014) in evaluating model fit for ordinal data within a measurement invariance framework, various estimators were employed, which also enhances research replication and sensitivity of the estimation method. However, in accordance with Finney et al. (2016), results from the WLSMV are reported since WLSMV is more appropriate with ordinal data (Sass et al., 2014; Finney et al., 2016).

A well-fitting CFA model was established in single-group analyses for all intervention sites and for females and males. Measurement invariance was then conducted using the MODEL option of the ANALYSIS command in Mplus with theta parameterization across intervention sites and across gender. Configural invariance was tested first, which also represented the baseline model for the subsequent and more restrictive models. To test the configural invariance, factor loadings and thresholds were freely estimated across groups, residual variances were fixed at 1 in all groups, and factor means were fixed at 0 in all groups. Using the marker variable approach to set the metric of the factors, factor variances were also freely estimated across groups. The test of metric invariance was conducted by constraining all factor loadings as equal across groups. Residual variances were fixed at 1 in one group and freely estimated in the other groups, and factor means were fixed at 0 in one group and freely estimated in the other groups. The first threshold of each item was held equal across groups; the second threshold of the marker variable was also held equal across groups. Factor variances were freely estimated across groups. Next, the test of scalar invariance constrained item thresholds as equal across the groups in addition to the previous configuration of the metric invariance.

Incremental validity of the READ was tested in two ways: (i) by using the popular regression approach to determine the additional contribution of the READ total score as well as the subscales in stepwise regression as separate predictors of depressive symptoms over and above the measures of perceived stress and symptoms of anxiety and (ii) by using the less common but efficient SEM approach, which accounts for measurement error, unlike the regression approach. Using the regression approach even when reliability seems adequate can produce misleading results (Wang and Eastwick, 2020). Established and widely accepted recommendations for testing incremental validity in SEM were followed (e.g., Wang and Eastwick, 2020). Although model fit indices may point to acceptable and adequate fit (MacCallum et al., 1996), a good model fit was evaluated with the following indices: standardized root mean square residual (SRMR) and RMSEA values less than 0.08 and values equal to or less than 0.06 (upper 90% CI close to or <0.08), respectively (Browne and Cudeck, 1993), and a comparative fit index (CFI) and a non-normed fit index [NNFI, aka Tucker–Lewis index (TLI)] greater than 0.95 (Hu and Bentler, 1999). CFI and TLI values between 0.90 and 0.95 and RMSEA between 0.08 and 0.06 are considered acceptable fit (Browne and Cudeck, 1993; Hu and Bentler, 1999). Since the chi-square test has been criticized for being too sensitive, a change of −0.010 or more in CFI and ≥0.015 in RMSEA or a change of ≥0.030 in SRMR was used as indicating non-invariance when testing metric invariance. For testing scalar invariance, we used the same changes in CFI and RMSEA, supplemented by a change of ≥0.010 in SRMR, as indicating non-invariance (Chen, 2007).

## Results

### Configural Invariance

The original five-factor structure of the READ fits reasonably well in all the sites (M1a–M1d; Table 1) as well as across females and males (L1a–L1b). The baseline configural invariance models across sites (M2) and across gender (L2) were all adequate as the equivalent five-factor model with identical factor patterns had acceptable fit across sites and gender. Table 2 presents the factor structure of the READ.

**Table 1:** Evaluations of multigroup confirmatory factor analysis measurement invariance (MGCFA MI; *N* = 1,546).

**Model**	**Type of test**	**Compared with**	**χ^**2**^**	***df***	**RMSEA**	**CFI**	**TLI**	**Δ*df***	**Δ*CFI***	**ΔRMSEA**	**Decision**
**Countries**											
M1a	Spain		744.492	340	0.055 [0.050–0.061]	0.940	0.933				
M1b	Iceland		902.955	340	0.066 [0.061–0.072]	0.949	0.944				
M1c	Italy		832.282	340	0.056 [0.051–0.061]	0.938	0.932				
M1d	Poland		838.391	340	0.068 [0.063–0.074]	0.932	0.924				
M2	Configural invariance		3,327.084	1,360	0.061 [0.059–0.064]	0.941	0.934				
M3	Metric invariance	M2	3,287.219	1,429	0.058 [0.056–0.061]	0.944	0.941	69	0.003	−0.003	Accept
M4	Scalar invariance	M3	3,943.741	1,666	0.060 [0.057–0.062]	0.932	0.938	237	−0.012	0.002	Accept
**Gender**											
L1a	Females		1,312.944	340	0.059 [0.056–0.062]	0.943	0.937				
L1b	Males		1,049.201	340	0.054 [0.051–0.058]	0.954	0.949				
L2	Configural invariance		2,368.613	680	0.057 [0.055–0.060]	0.948	0.942				
L3	Metric invariance	L2	2,314.938	703	0.055 [0.052–0.057]	0.950	0.946	23	0.002	−0.002	Accept
L4	Scalar invariance	L3	2,420.840	782	0.052 [0.050–0.055]	0.949	0.951	79	−0.001	−0.003	Accept

*χ^2^, chi-square statistic; df, degrees of freedom; RMSEA, root mean square error of approximation; CFI, comparative fit index; TLI, Tucker–Lewis index; Δ, change in statistical values*.

**Table 2:** Factor structure of the Resilience Scale for Adolescents (READ; *N* = 1,546).

**Item**	**PC**	**SC**	**SS**	**SR**	**FC**
READ1	0.56				
READ4	0.73				
READ7	0.67				
READ12	0.34				
READ17	0.70				
READ20	0.61				
READ23	0.67				
READ26	0.67				
READ6		0.70			
READ11		0.71			
READ16		0.70			
READ22		0.72			
READ25		0.65			
READ2			0.64		
READ8			0.56		
READ13			0.63		
READ18			0.60		
READ3				0.73	
READ9				0.56	
READ14				0.72	
READ19				0.77	
READ28				0.72	
READ5					0.72
READ10					0.79
READ15					0.77
READ21					0.72
READ24					0.84
READ27					0.75

*PC, personal competence; SC, social competence; SS, structured style; SR, social resources; FC, family cohesion*.

### Metric Invariance

The baseline configural invariance models across sites (M2) and across gender (L2) were compared to the next level of invariance model constraining the factor loadings equally across sites (M3) and gender (L3), thus testing the important assumption of metric invariance. There was no worsening in fit as the fit indices showed improvement over the configural invariance. Metric invariance across sites and gender was thus achieved.

### Scalar Invariance

The fit of the model constraining item thresholds as equal across sites (M4) and across gender (L4) was not worse than the model allowing different item thresholds across sites (M3) and across gender (L3), even though the RMSEA, CFI, and TLI showed a slight decline in fit. The worsening in fit was minor with regard to the ΔCFI of −0.012, and the ΔRMSEA did not meet the threshold to reject. Support for scalar invariance across sites and gender was thus achieved.

### Convergent and Discriminant Validity of the READ and Subscales

As expected, the READ total score correlated significantly positively with measures of well-being (*r* = 0.72, *p* < 0.001) and health-related quality of life (*r* = 0.73, *p* < 0.001), but significantly negatively with perceived stress score (*r* = −0.53, *p* < 0.01), symptoms of anxiety (*r* = −0.43, *p* < 0.01), and depression (*r* = −0.53, *p* < 0.001). The subscales of the READ also correlated significantly positively with mental well-being (ranging from *r* = 0.54 to 0.70) and health-related quality of life (*r* = 0.49–0.72). Conversely, the READ subscales correlated significantly negatively with perceived stress (*r* = −0.34 to −0.57), symptoms of anxiety (*r* = −0.26 to −0.49), and depression (*r* = −0.37 to −0.45).

### Incremental Validity of the READ and Subscales

Incremental validity using the regression approach was assessed by identifying whether the increment in certainty of the prediction (Δ*R*^2^) was significant when the resilience total score or a subscale was included in the model. In step 1, perceived stress (standardized: β = 0.24, SE = 0.04, *t* = 11.48, *p* < 0.001) and symptoms of anxiety (β = 0.60, SE = 0.02, *t* = 28.90, *p* < 0.001) significantly positively predicted depressive symptoms (*R*^2^ = 0.58). The resilience total score in step 2 predicted depressive symptoms (β = −0.21, SE = 0.17, *t* = −10.21, *p* < 0.001), accounting for additional variance (Δ*R*^2^ = 0.03). The results from substituting separate subscales in step 2 were as follows: personal competence: β = −0.19, SE = 0.16, *t* = −8.68, *p* < 0.001, Δ*R*^2^ = 0.02; social competence: β = −0.09, SE = 0.12, *t* = −4.68, *p* < 0.001, Δ*R*^2^ = 0.01; structured style: β = −0.15, SE = 0.12, *t* = −8.14, *p* < 0.001, Δ*R*^2^ = 0.02; social resources; β = −0.13, SE = 0.14, *t* = −6.95, *p* < 0.001, Δ*R*^2^ = 0.01; and family cohesion: β = −0.19, SE = 0.12, *t* = −9.93, *p* < 0.001, Δ*R*^2^ = 0.03.

In the SEM approach to testing incremental validity, a minimum requirement was to establish empirical evidence for construct separability between resilience and the other covariates (perceived stress and anxiety symptoms) in a CFA that compares a unifactorial model (resilience, perceived stress, and anxiety symptoms are not separate constructs) to a three-factor model (resilience, perceived stress, and anxiety symptoms are separate constructs). The unifactorial model failed to reach acceptable fit (χ^2^ = 2,164.739, *df* = 102, *p* < 0.001; SRMR = 0.09, RMSEA = 0.12, 90% CI = 0.110–0.119, CFI = 0.70, TLI = 0.65), while the three-factor model was an improvement (χ^2^ = 584.081, *df* = 100, *p* < 0.001; SRMR = 0.05, RMSEA = 0.06, 90% CI = 0.052–0.061, CFI = 0.93, TLI = 0.92). After convergence on better and meaningful fit, factor correlations were inspected. Inspection of the factor correlations showed that stress correlated with anxiety on the threshold of large correlation (*r* = 0.60, *p* < 0.001), but most importantly, resilience significantly negatively correlated with stress (*r* = −0.74, *p* < 0.001) and anxiety symptoms (*r* = −0.52, *p* < 0.001), thus providing strong evidence that resilience was a separate construct from perceived stress and anxiety symptoms. Incremental validity in the SEM approach (contained in the Supplementary Material, pp. 4–9; Supplementary Figures 1–6) was evidenced by a significant path coefficient from the resilience total score or a subscale to depressive symptoms over and above the perceived stress and symptoms of anxiety for READ total score (β = −0.21, *p* < 0.01), personal competence (β = −0.71, *p* < 0.05), structured style (β = −0.15, *p* < 0.05), social resources (β = −0.13, *p* < 0.05), and family cohesion (β = −0.19, *p* < 0.001), but not social competence (β = −0.04, *p* = 0.572).

### Mean Differences in Total Scores and Subscales

Table 3 presents the means, standard deviations, and reliability estimates of the READ with subscales. Potential gender differences were explored with independent *t* tests. In the Basque Country, no gender differences were found on the resilience total score and subscales. In the sample from Iceland, females scored significantly lower on the resilience total score (mean difference, MD = −0.164, 95% CI = −0.278 to −0.050), with a small effect size (*d* = −0.288), personal competence (MD = −0.331, 95% CI = −0.463 to −0.200), with a medium effect size (*d* = −0.526), and social competence (MD = −0.207, 95% CI = −0.354 to −0.060), with a small effect size (*d* = −0.295). In the sample from Italy, gender differences were found only for social resources, with females scoring higher than males (MD = 0.172, 95% CI = 0.048–0.295), with a small effect size (*d* = 0.261). In the sample from Poland, gender differences were found only for personal competence, with females scoring lower than males (MD = −0.197, 95% CI = −0.345 to −0.048), with a small effect size (*d* = −0.316). In the combined data, females scored lower than males on personal competence (MD = −0.181, 95% CI = −0.246 to −0.115), with a small effect size (*d* = −0.283), but scored higher than males on social resources (MD = 0.071, 95% CI = 0.006–0.137), with a small effect size (*d* = 0.108).

**Table 3:** Means, standard deviations, and reliability estimates of the Resilience Scale for Adults (RSA) with subscales across all countries (*N* = 1,546).

		**Mean (SD)**				**Coefficient alpha (*****α*****)**				**Coefficient omega (*****ω*****)**			
		**Spain (*n* = 391)**	**Iceland (*n* = 379)**	**Italy (*n* = 460)**	**Poland (*n* = 316)**	**Basque**	**Iceland**	**Italy**	**Poland**	**Spain**	**Iceland**	**Italy**	**Poland**
1	Perceived stress	5.34 (2.76)	5.62 (2.85)	5.97 (2.86)	5.79 (2.81)	0.60	0.56	0.62	0.59				
2	Anxiety symptoms	5.15 (4.07)	4.68 (4.22)	6.27 (4.30)	5.40 (4.73)	0.80	0.85	0.80	0.87				
3	Depressive symptoms	5.46 (4.28)	5.89 (4.80)	6.51 (4.49)	7.56 (5.18)	0.77	0.84	0.72	0.80				
4	Mental well-being	54.18 (7.10)	51.54 (8.16)	48.63 (7.19)	50.59 (8.32)	0.83	0.88	0.79	0.85				
5	Quality of life	74.53 (14.29)	71.00 (15.37)	67.07 (13.89)	64.50 (15.77)	0.60	0.58	0.49	0.55				
6	READ total	4.01 (0.51)	3.91 (0.55)	3.70 (0.54)	3.73 (0.59)	0.91	0.94	0.91	0.93	0.92	0.94	0.92	0.94
7	Personal competence	3.84 (0.61)	3.72 (0.64)	3.55 (0.64)	3.60 (0.65)	0.76	0.84	0.78	0.81	0.76	0.84	0.79	0.81
8	Social competence	3.94 (0.65)	3.76 (0.71)	3.68 (0.71)	3.73 (0.75)	0.76	0.78	0.77	0.80	0.76	0.78	0.77	0.81
9	Structured style	3.68 (0.70)	3.63 (0.73)	3.30 (0.71)	3.34 (0.68)	0.64	0.71	0.59	0.60	0.66	0.73	0.59	0.58
10	Social resources	4.39 (0.54)	4.30 (0.57)	4.06 (0.64)	4.03 (0.75)	0.71	0.78	0.71	0.83	0.71	0.78	0.72	0.83
11	Family cohesion	4.20 (0.66)	4.14 (0.69)	3.88 (0.75)	3.92 (0.73)	0.84	0.87	0.85	0.86	0.84	0.88	0.86	0.86

A one-way multivariate ANOVA (MANOVA) was conducted, which showed significant between-country differences on the resilience total score and subscales: Wilks' lambda, λ = 0.910 [*F*_(15, 3722)_ = 8.643, *p* < 0.001, partial η^2^ = 0.031]. Follow-up univariate ANOVA correcting for alpha at *p* < 0.01 showed significant effect on the resilience total score [*F*_(3, 1352)_ = 24.77, *p* < 0.001, partial η^2^ = 0.05], personal competence [*F*_(3, 1352)_ = 14.05, *p* < 0.001, partial η^2^ = 0.03], social competence [*F*_(3, 1352)_ = 8.97, *p* < 0.001, partial η^2^ = 0.02], structured style [*F*_(3, 1352)_ = 26.27, *p* < 0.001, partial η^2^ = 0.06], social resources [*F*_(3, 1352)_ = 26.82, *p* < 0.001, partial η^2^ = 0.06], and family cohesion [*F*_(3, 1352)_ = 17.74, *p* < 0.001, partial η^2^ = 0.04]. *Post hoc* pairwise comparisons with Scheffé revealed significant differences between countries on the resilience total score and subscales contained in the Supplementary Material (pp. 2–3; Supplementary Table 1).

## Discussion

The goal of this study was to investigate the operationalization of resilience by the Resilience Scale for Adolescents (READ) in samples across the Basque Country (Spain), Reykjavík (Iceland), Trentino (Italy), and Lower Silesia (Poland) after the translation of the instrument as part of the UPRIGHT project (Las Hayas et al., 2019). The results supported the 28-item five-factor model of the READ as well as its psychometric properties, internal consistency, and construct and incremental validity. Measurement invariance across the intervention sites and gender was supported from configural to scalar invariance, thus allowing meaningful scale mean comparisons between the intervention sites. The original five-factor structure of the READ reproduced well in early adolescent samples across the different sites. Fit indices in terms of the RMSEA, which penalizes for model misspecification in relation to model complexity and sample size, were slightly above the threshold for a good model fit in the samples from Iceland (RMSEA = 0.066) and Poland (RMSEA = 0.068). Since the upper limit of the 90% CI did not exceed 0.08 and other fit indices pointed to adequate model fit, these models were retained (Browne and Cudeck, 1993; MacCallum et al., 1996; Hu and Bentler, 1999).

Support was found for configural invariance when the measurement models of the READ for the separate intervention sites were stacked on each other as well as stacking together separate models from females and males. Practically, this means that the operationalization of resilience by the READ is measured equivalently in the samples across all pilot sites. This finding supports previous findings by Kelly et al. (2017) in an Irish adolescent sample. Most importantly, and similar to the study by Askeland et al. (2019), support was found for metric invariance, which in the context of UPRIGHT means that resilience manifests similarly and that all adolescents have understood and interpreted the READ scale items equivalently. Therefore, simple regression analyses based on raw scores may be used to predict comparable changes in criterion-related outcome variables across the intervention sites. This is not surprising since the READ was developed based on the adult version RSA using available international empirical evidence at the time, supplemented by the overarching theoretical framework in resilience research (Hjemdal et al., 2001).

The previous study by Askeland et al. (2019) did not find full support for scalar invariance. Our findings supported invariance in the item threshold parameters (scalar invariance) that link the underlying continuous latent resilience construct to the observed response categories of the READ items. In practice, this means that the adolescent samples from the different intervention sites use the Likert response format of the READ items in the same way; hence, an adolescent from the different intervention sites with the same access and availability of protective factors related to resilience should obtain the same score on the READ items. This important finding reinforces the cross-cultural validity of the READ as a valid and reliable scale that measures protective factors related to resilience. The UPRIGHT project can therefore analyze meaningful and comparable scale means across pilot sites. It is important to note that the study by Askeland et al. (2019) included a slightly modified READ and that the authors concluded that their five-factor model achieved a better fit and theoretical compliance with resilience than other competing models. It would have been interesting to know from the study by Askeland et al. (2019) how the methodological concerns (e.g., positivity and acquiescence bias and ordinality of scale items) mentioned in the present study were treated in combination with their theoretical and conceptual concerns. It is recommended that future studies of the READ's construct validation and psychometric properties proceed in a confirmatory framework, address positivity and acquiescence biases, as well as take the ordinality of READ's data into account when continuous estimation methods produce less than adequate fit.

Group mean differences were observed across the intervention sites (see Supplementary Material, pp. 2–3; Supplementary Table 1) and across gender. When investigating group mean differences in the resilience total score, adolescent samples in the Basque Country scored significantly higher than those in Italy and Poland. Similarly, adolescent samples in Iceland scored significantly higher than adolescent samples in Italy and Poland. These are interesting differences for the UPRIGHT project evaluation and planning. Consistent with previous studies (Hjemdal et al., 2006; Kelly et al., 2017; e.g., Askeland et al., 2019) that reported higher mean scores on personal competence for males and social resources for females, our findings also indicated that males in Iceland and Poland (and in the combined sample) scored significantly higher on the personal competence subscale, whereas females in Italy and in the combined sample scored higher on the social resources subscale. Hjemdal et al. (2006) indicated that females are more skilled and socially sensitive in accessing social support and resources, whereas males report higher self-esteem, confidence, assertiveness, and feel personally competent, thus developing their levels of resilience through internal (personal) resources and females through external (social) resources. These findings are similar to the gender differences in the adult resilience version (Hjemdal et al., 2001; Friborg et al., 2003; Anyan et al., 2019). Anyan et al. (2019) concluded that the observed gender differences may be scale-specific since the overall levels of resilience were not related to gender differences. While prior studies have not found any gender differences on the social competence subscale, it was found in this study that males in Iceland scored significantly higher on the social competence subscale. Thus, our initial interpretation is that early adolescent males in Iceland may show higher hedonic and gregarious tendencies, experience positive emotions in interpersonal interactions, and are more flexible and outgoing, which is an interesting cultural difference that requires further exploration in future studies.

UPRIGHT implements a resilience-based intervention to promote adaptive mental health in adolescents. It is therefore important to observe that the READ and its subscales correlated significantly positively with health-related quality of life and adaptive aspects of mental health, but significantly negatively with perceived stress, anxiety, and depressive symptoms. Additionally, in the incremental validity analyses, when the resilience total score and the subscales were substituted in separate hierarchical analyses in step 2, they all explained some variance in depressive symptoms over and above the perceived stress and anxiety symptoms. A SEM approach to incremental validity analyses brings the findings into further relief as it was found that the resilience total score as well as the subscales, except social competence, significantly negatively predicted depressive symptoms. Our findings corroborate the findings from previous studies using the READ to indicate that the READ proves to be a valid and reliable instrument to measure protective factors of resilience that may promote well-being and positive mental health against exposure to negative or stressful life events, experience of bullying, anxiety, and depressive symptoms (Anyan and Hjemdal, 2016, 2018; Kelly et al., 2017; Anyan et al., 2018; Moksnes and Haugan, 2018; Askeland et al., 2019). Higher scores on the READ were found to be associated with sense of coherence, self-esteem (Moksnes and Haugan, 2018), safe neighborhood, enjoying family life, and informal help-seeking (Kelly et al., 2017) as well as participation in hobbies and team sports or athletic clubs and active engagement in social interaction (Hjemdal et al., 2006), which are all relevant positive personal characteristics and psychosocial factors involved in resilience outcomes and processes. It is the interplay of these protective factors in a complex and dynamic process with the environment that contributes to positive adaptation and development. There were limitations to the present study, some of which offer potential avenues for further investigations. The age of the participants was between 12 and 14 years, which only allows for validating the READ in early adolescence. Future studies of the entire adolescent developmental span are needed to account for within-person changes over time since resilience is conceptualized as both a process and an outcome. READ will benefit from future studies testing its reliability and validity in Asian, African, and other cultures.

## Conclusions

In conclusion, the 28-item five-factor model of READ found support as a valid and reliable instrument to measure central protective factors for resilience including personal positive characteristics, family environment, and external social support system. Robust evidence for the psychometric properties and construct validity of the READ was found through structural validity using measurement invariance and also through convergent, discriminant, and incremental validity tests. Some prior studies validating the READ have reported inadmissible factor solutions and, in some cases, low indicator loadings, resulting in revised factor structures. These problems may be the result of (i) not remedying positivity and acquiescence biases and (ii) ignoring the ordinality of the scale items. Brown (2015) discusses the consequences for ignoring ordinality of scale items, including incorrect parameter estimates and test statistics, factors that are artifacts of item extremeness, inconsistent factor structures, and, finally, attenuated relationship between factors, problems that typically characterize most of the previous studies.

We conclude with the following recommendations for future investigations using the READ. When continuous estimation methods report less than adequate fit for the READ factor structure, investigators should explore categorical estimation, taking the ordinality of the READ data and the potential effect of positivity and pronounced acquiescence bias in younger children and adolescents into account. Results from categorical estimation should be reported when there are differences in using continuous and categorical estimation. Future investigators seeking to validate the READ should proceed in a confirmatory framework. These recommendations can advance assessment science and practice with regard to measuring resilience across different groups, contexts, and cultures. The analysis procedure highlighted in our recommendations also has relevance for and may generalize to other measures that show the same pattern of data as the READ.

## Data Availability Statement

The raw data supporting the conclusions of this article will be made available by the authors, without undue reservation.

## Ethics Statement

Ethical clearance was obtained by individual countries based on local regulations as part of the UPRIGHT EU HORIZON 2020 Project. Written informed consent to participate in this study was provided by the participants' legal guardian/next of kin.

## On Behalf of Upright Consortium

Kronikgune (Spain)

Esteban de Manuel, Ane Fullaondo, Irantzu Izco-Basurko, Maider Mateo, Igor Larranga

Osakidetza (Spain)

Ana González-Pinto, Inaki Zorilla, Patricia Pérez Martínez, Itziar Vegara, Javier Mar

UP/EHV (Spain)

Jessica Fernández Sevillano

FBK (Italy)

Sara Carbone, Silvia Rizzi, Valeria Donisi

DOHI (Iceland)

Solveig Karlsdottir

AU (Denmark)

Mette Marie Ledertoug, Hans Henrik Knoop, Louise Tidman

UoI (Iceland)

Ingibjorg V. Kaldalons, Bryndis Jona Jonsdottir, Alda Ingibergsdottir, Hrefna Palsdottir, Unnur B. Arnfjord

## Author Contributions

FA conducted data analyses, wrote the first draft of the manuscript, and continuously revised and developed the final version based on feedback received. RM and OH provided substantial feedback on the manuscript, data analyses, and revisions. CL, SG, IM, DG, NG, AK, AZ, and AO provided the data for the study and substantial feedback on the manuscript and revisions. All authors have read and approved the manuscript.

## Conflict of Interest

The authors declare that the research was conducted in the absence of any commercial or financial relationships that could be construed as a potential conflict of interest.

## Footnotes

^1^For ease of referencing, we use country-level names (e.g., Spain or Iceland). Denmark is not included in this study because the READ has already been validated in a Danish-speaking sample.
