# Supplementary material for: Measuring Resilience Across Participating Regions in the UPRIGHT EU Horizon 2020 Project: Factor Structure and Psychometric Properties of the Resilience Scale for Adolescents
Source: Front Psychol. 2021 Feb 17;12:629357. doi: 10.3389/fpsyg.2021.629357 (PMC7928283; doi:10.3389/fpsyg.2021.629357)

Supplementary Material

Article Title: Measuring resilience across participating regions in the UPRIGHT EU Horizon 2020 project: Factor structure and psychometric properties of the Resilience Scale for Adolescents (READ).

Journal name: Frontiers in Psychology (Quantitative Psychology and Measurement)

Frederick Anyan[1], Roxanna Morote[1], Carlota Las Hayas[2], Silvia Gabrielli[3], Iwona Mazur[4,9], Dora Gudrun Gudmundsdottir[5], Nerea González[2,6], Anna Królicka-Deregowska[4], Antoni Zwiefka[7], Anna S. Olafsdottir[8], Odin Hjemdal[1], on behalf of UPRIGHT

[1]Norwegian University of Science and Technology, Trondheim, Norway
[2]Kronikgune Institute for Health Services Research, Basque Country, Spain
[3]Bruno Kessler Foundation, Trento, Italy
[4]Daily Centre for Psychiatry and Speech Disorders, Wrocław, Poland
[5]Directorate of Health, Reykjavík, Iceland.
[6]Osakidetza Basque Health Service, Barrualde-Galdakao Integrated Health Organisation; REDISSEC (Health Services Research on Chronic Patients Network), Basque Country, Spain
[7]Lower Silesian Voivodeship Marshal Office
[8]University of Iceland, School of Education, Reykjavik, Iceland
[9]Wroclaw Medical University, Wrocław, Poland

Correspondence should be sent to

Frederick Anyan, PhD

Department of Psychology, Norwegian University of Science and Technology, NO – 7491. Trondheim, Norway.

Email: frederick.anyan@ntnu.no

Table S1: Multiple Comparisons using Scheffe (*N* = 1 546)

Dependent Variable			Mean Difference	Std. Error	Sig.	95% CI	
Resilience total score	Spain	Iceland	0.10	0.042	0.120	-0.02	0.22
		Italy	.31*	0.041	0.000	0.19	0.42
		Poland	.28*	0.044	0.000	0.16	0.40
	Iceland	Spain	-0.10	0.042	0.120	-0.22	0.02
		Italy	.21*	0.040	0.000	0.09	0.32
		Poland	.18*	0.044	0.001	0.06	0.30
	Italy	Spain	-.31*	0.041	0.000	-0.42	-0.19
		Iceland	-.21*	0.040	0.000	-0.32	-0.09
		Poland	-0.03	0.043	0.937	-0.15	0.09
	Poland	Spain	-.28*	0.044	0.000	-0.40	-0.16
		Iceland	-.18*	0.044	0.001	-0.30	-0.06
		Italy	0.03	0.043	0.937	-0.09	0.15
Personal competence	Spain	Iceland	0.12	0.048	0.100	-0.01	0.26
		Italy	.28*	0.047	0.000	0.15	0.41
		Poland	.24*	0.051	0.000	0.10	0.38
	Iceland	Spain	-0.12	0.048	0.100	-0.26	0.01
		Italy	.16*	0.047	0.007	0.03	0.29
		Poland	0.12	0.051	0.142	-0.02	0.26

	Italy	Spain	-.28*	0.047	0.000	-0.41	-0.15
		Iceland	-.16*	0.047	0.007	-0.29	-0.03
		Poland	-0.04	0.050	0.856	-0.18	0.10
	Poland	Spain	-.24*	0.051	0.000	-0.38	-0.10
		Iceland	-0.12	0.051	0.142	-0.26	0.02
		Italy	0.04	0.050	0.856	-0.10	0.18
Social competence	Spain	Iceland	.18*	0.054	0.012	0.03	0.33
		Italy	.26*	0.052	0.000	0.11	0.41
		Poland	.22*	0.057	0.003	0.06	0.38
	Iceland	Spain	-.18*	0.054	0.012	-0.33	-0.03
		Italy	0.08	0.052	0.505	-0.07	0.23
		Poland	0.04	0.057	0.935	-0.12	0.20
	Italy	Spain	-.26*	0.052	0.000	-0.41	-0.11
		Iceland	-0.08	0.052	0.505	-0.23	0.07
		Poland	-0.04	0.056	0.900	-0.20	0.11
	Poland	Spain	-.22*	0.057	0.003	-0.38	-0.06
		Iceland	-0.04	0.057	0.935	-0.20	0.12
		Italy	0.04	0.056	0.900	-0.11	0.20
Structured style	Spain	Iceland	0.05	0.054	0.804	-0.10	0.20
		Italy	.38*	0.052	0.000	0.24	0.53
		Poland	.34*	0.057	0.000	0.18	0.50

	Iceland	Spain	-0.05	0.054	0.804	-0.20	0.10
		Italy	.33*	0.052	0.000	0.18	0.47
		Poland	.28*	0.057	0.000	0.13	0.44
	Italy	Spain	-.38*	0.052	0.000	-0.53	-0.24
		Iceland	-.33*	0.052	0.000	-0.47	-0.18
		Poland	-0.04	0.056	0.891	-0.20	0.11
	Poland	Spain	-.34*	0.057	0.000	-0.50	-0.18
		Iceland	-.28*	0.057	0.000	-0.44	-0.13
		Italy	0.04	0.056	0.891	-0.11	0.20
Social resources	Spain	Iceland	0.08	0.047	0.406	-0.05	0.21
		Italy	.33*	0.046	0.000	0.20	0.46
		Poland	.36*	0.051	0.000	0.22	0.50
	Iceland	Spain	-0.08	0.047	0.406	-0.21	0.05
		Italy	.25*	0.046	0.000	0.12	0.37
		Poland	.28*	0.050	0.000	0.14	0.42
	Italy	Spain	-.33*	0.046	0.000	-0.46	-0.20
		Iceland	-.25*	0.046	0.000	-0.37	-0.12
		Poland	0.03	0.049	0.942	-0.11	0.17
	Poland	Spain	-.36*	0.051	0.000	-0.50	-0.22
		Iceland	-.28*	0.050	0.000	-0.42	-0.14
		Italy	-0.03	0.049	0.942	-0.17	0.11

Family cohesion	Spain	Iceland	0.06	0.054	0.762	-0.09	0.21
		Italy	.32*	0.053	0.000	0.17	0.47
		Poland	.29*	0.057	0.000	0.13	0.45
	Iceland	Spain	-0.06	0.054	0.762	-0.21	0.09
		Italy	.26*	0.052	0.000	0.12	0.41
		Poland	.23*	0.057	0.001	0.07	0.39
	Italy	Spain	-.32*	0.053	0.000	-0.47	-0.17
		Iceland	-.26*	0.052	0.000	-0.41	-0.12
		Poland	-0.03	0.056	0.953	-0.19	0.12
	Poland	Spain	-.29*	0.057	0.000	-0.45	-0.13
		Iceland	-.23*	0.057	0.001	-0.39	-0.07
		Italy	0.03	0.056	0.953	-0.12	0.19

Figure S1: Resilience total score path model with only significant paths included to preserve readability of the figure (*N* = 1 546)

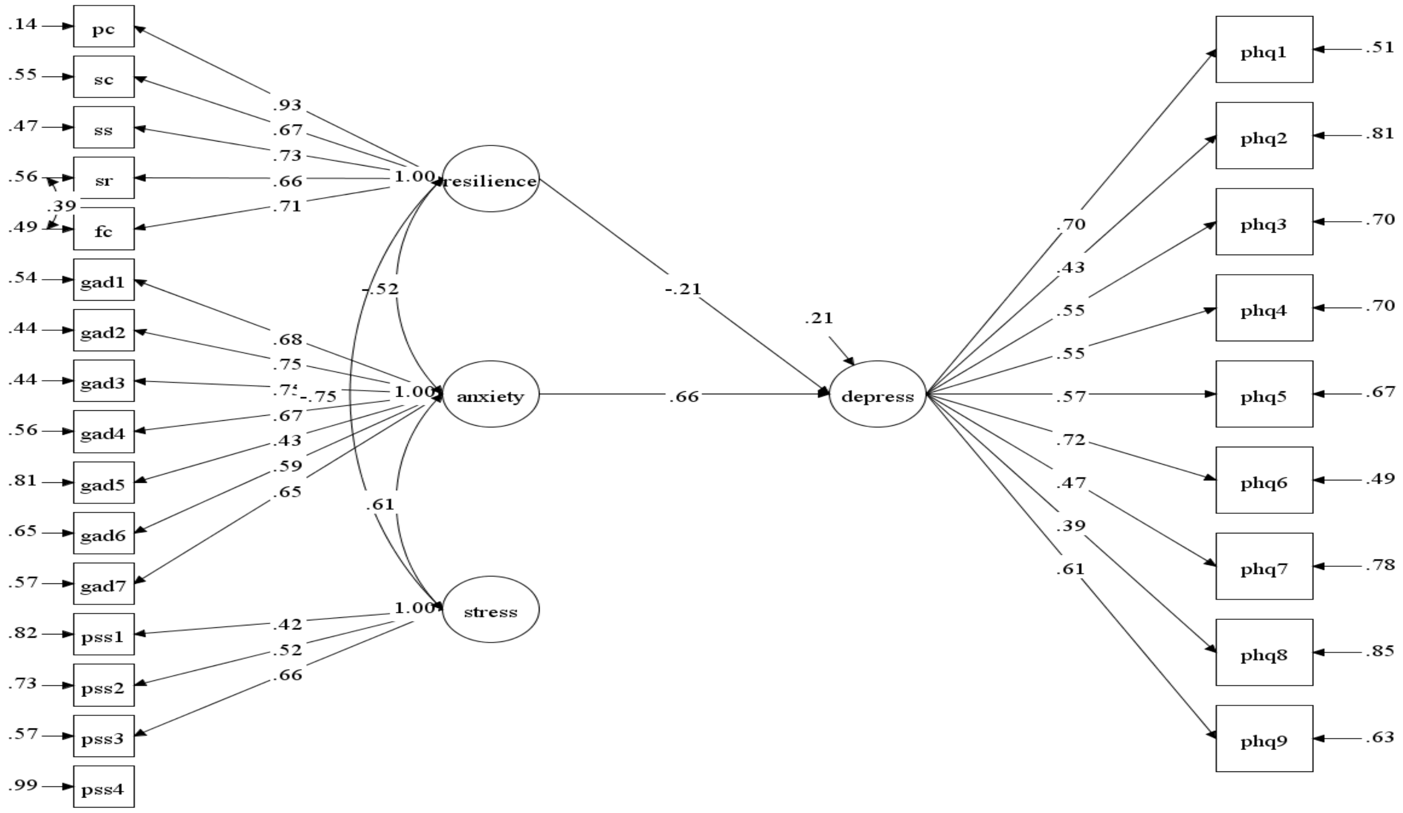

We explored a model with resilience total score where the subscales were used as factor indicators.

Figure S2: Personal competence (PC) path model with only significant paths included to preserve readability of the figure (N = 1 546)

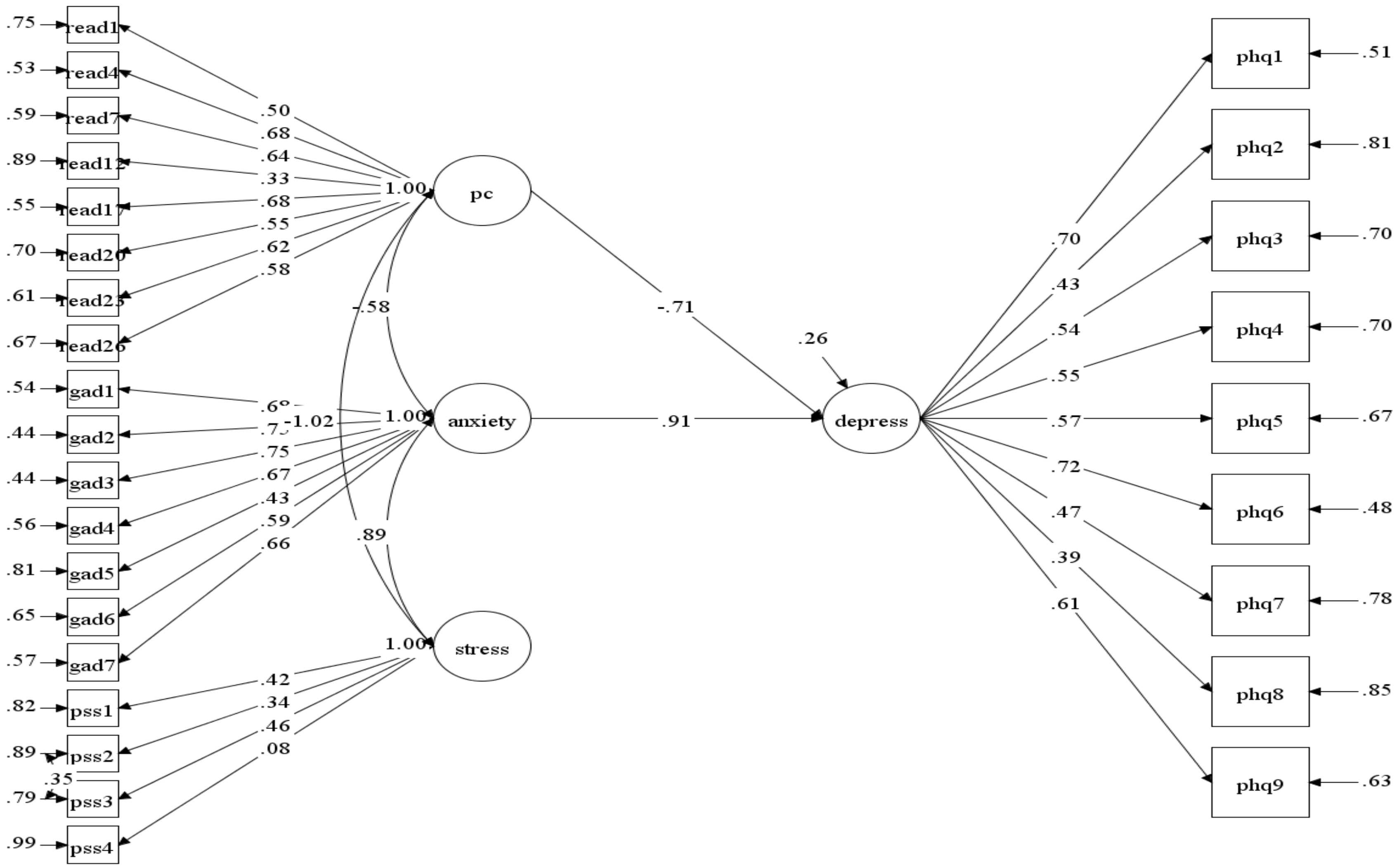

Figure S3: Social competence (SC) path model with only significant paths included to preserve readability of the figure (N = 1 546)

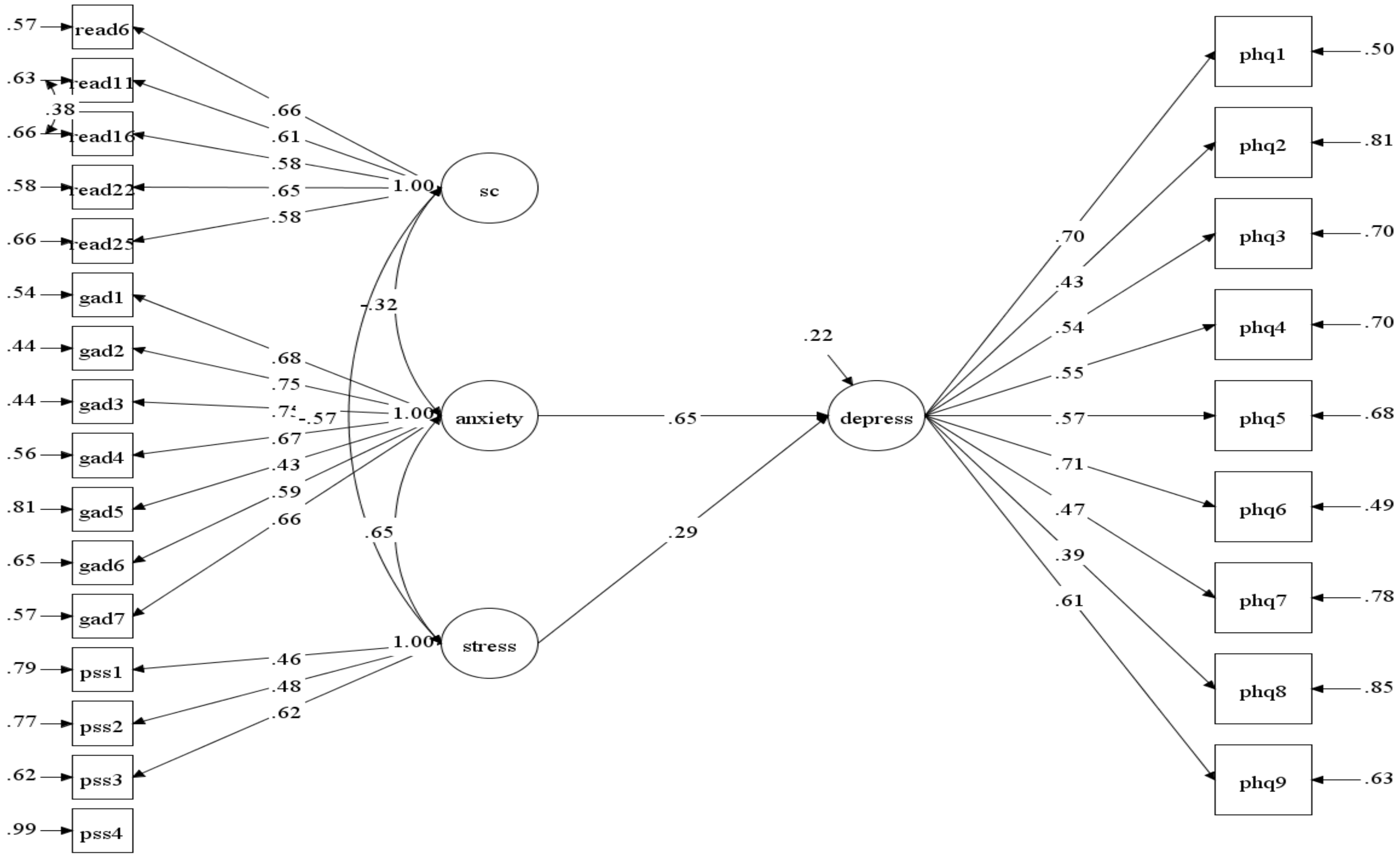

Figure S4: Structured style (SS) path model with only significant paths included to preserve readability of the figure (N = 1 546)

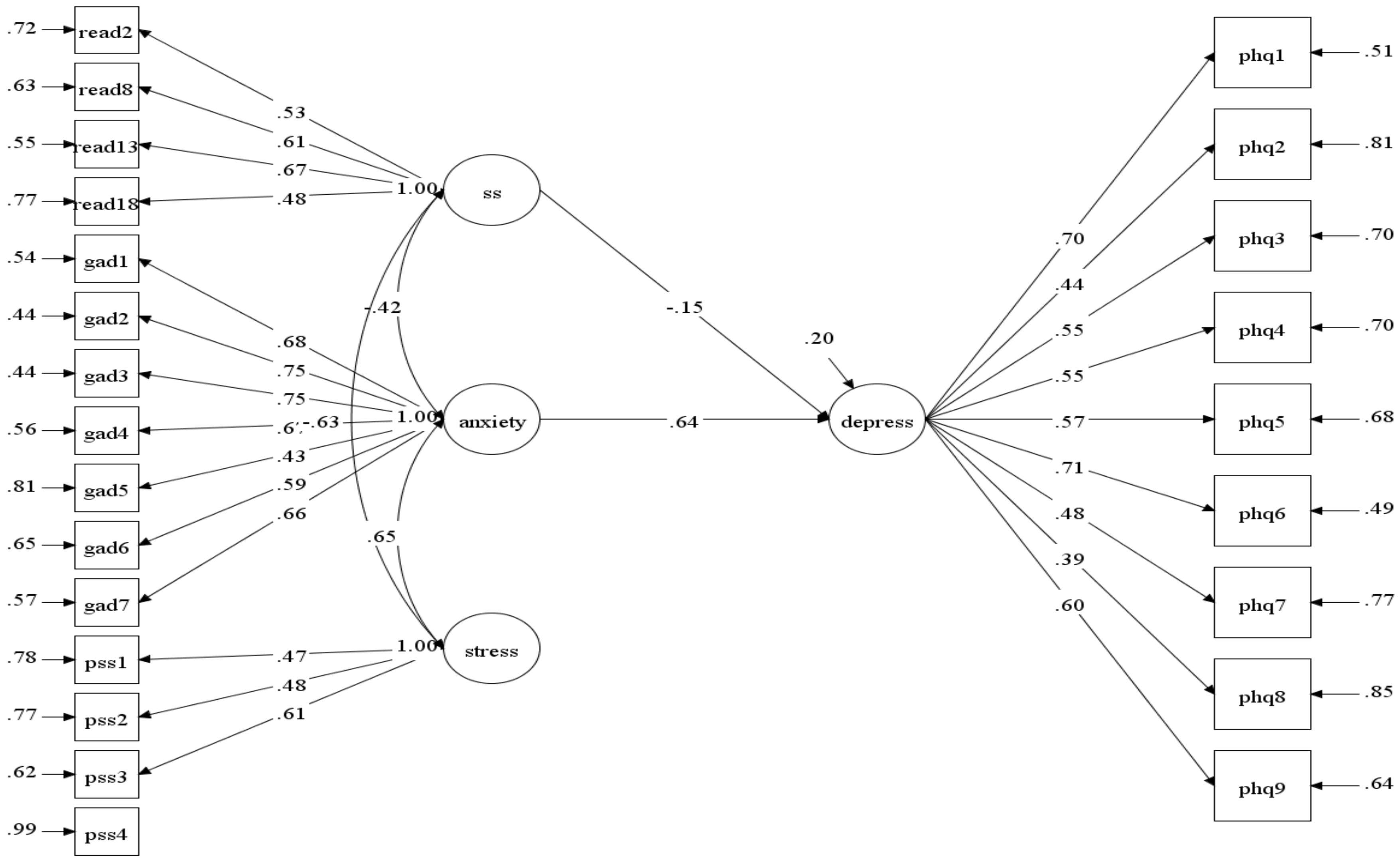

Figure S5: Social resources (SR) path model with only significant paths included to preserve readability of the figure (N = 1 546)

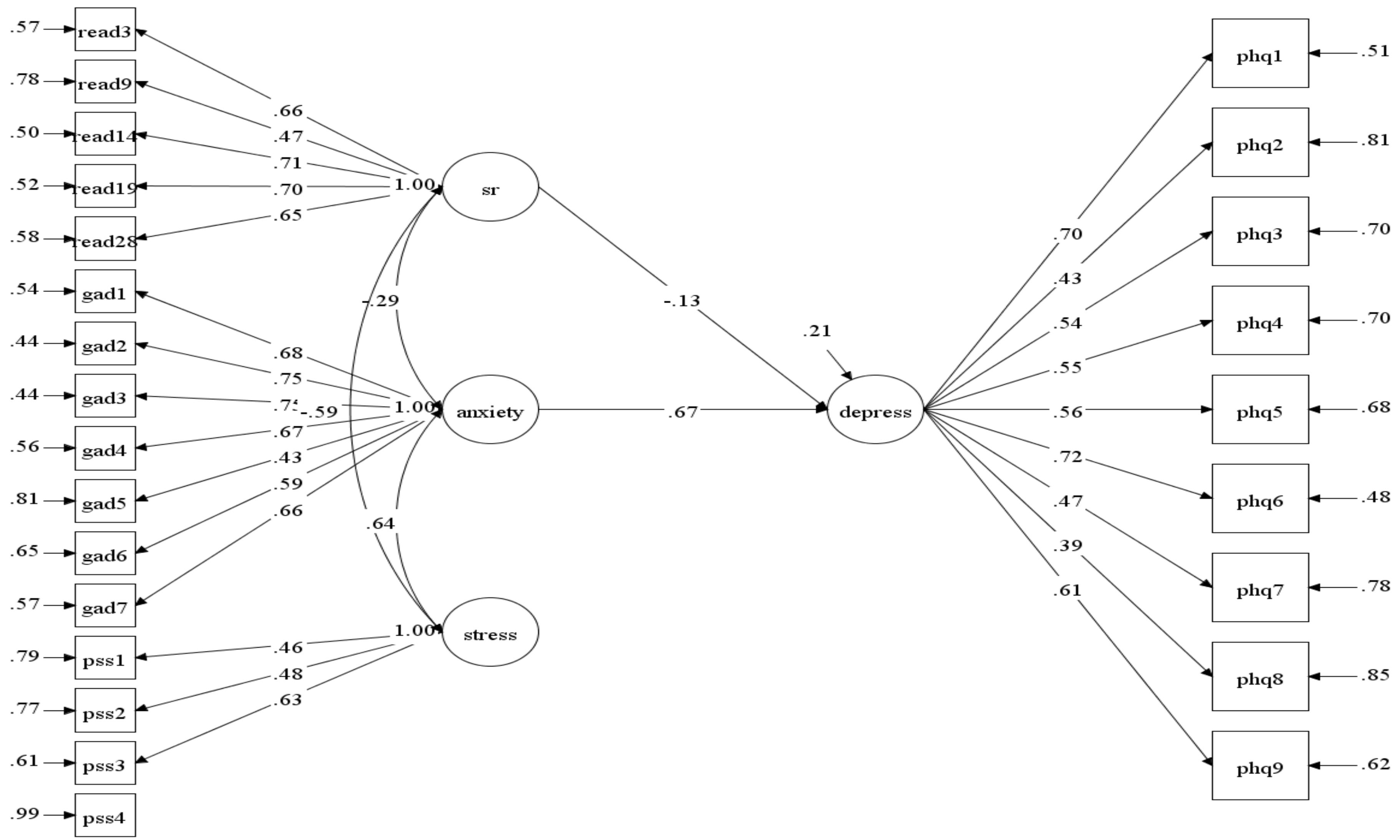

Figure S6: Family cohesion (FC) path model with only significant paths included to preserve readability of the figure (N = 1 546)

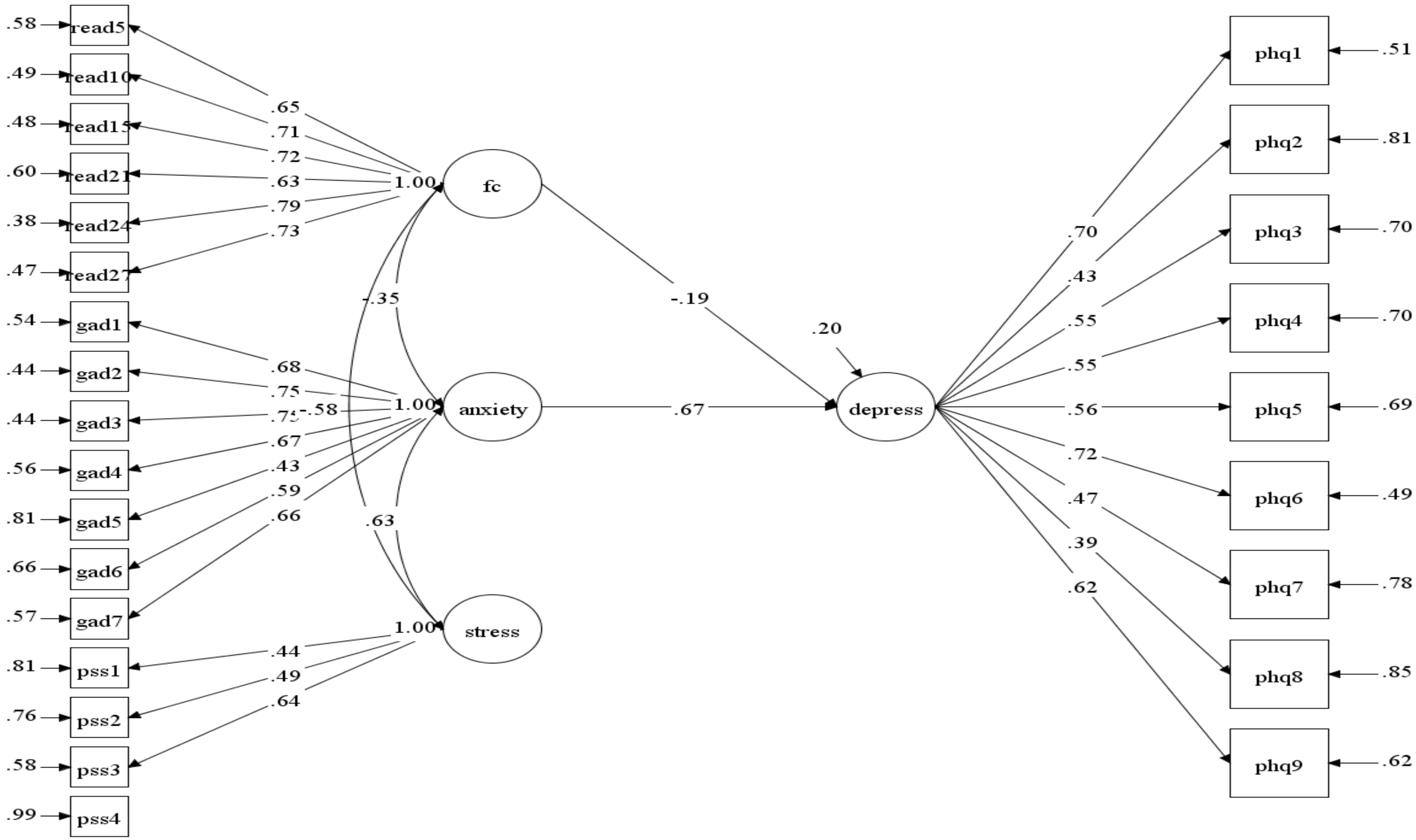

Supplement: Supplementary file 1 [file Data_Sheet_1.PDF]
